# 2-Benzoyl-2*H*-1,4-benzothia­zin-3(4*H*)-one

**DOI:** 10.1107/S1600536810029582

**Published:** 2010-07-31

**Authors:** Durre Shahwar, M. Nawaz Tahir, Naeem Ahmad, Muhammad Asam Raza, Muhammad Akmal Khan

**Affiliations:** a Department of Chemistry, Government College University, Lahore, Pakistan; b Department of Physics, University of Sargodha, Sargodha, Pakistan

## Abstract

In the title compound, C_15_H_11_NO_2_S, the dihedral angle between the aromatic rings is 80.35 (7)°. The heterocyclic six-membered ring is not planar: the puckering parameters of this ring are *Q* = 0.5308 (15) Å, θ = 63.11 (18) and ϕ = 23.5 (2)°. The mol­ecules are linked into inversion dimers with *R*_2_^2^(8) ring motifs by pairs of N—H⋯O hydrogen bonds. The dimers are inter­linked into polymeric sheets extending parallel to the *bc* plane by C—H⋯O hydrogen bonds, generating *R*_2_^1^(7) ring motifs. π–π inter­actions occur between the benzoyl phenyl rings with centroid–centroid separations of 3.9187 (15) Å.

## Related literature

For puckering parameters, see: Cremer & Pople (1975 ▶). For the synthesis and anti­microbial activity of benzimidazole derivatives, see: Güven *et al.* (2007 ▶): Nofal *et al.* (2002 ▶). For related structures, see: Beryozkina *et al.* (2004 ▶): Kumaradhas & Nirmala (1997 ▶): Zhang *et al.* (2008 ▶). For graph-set notation, see: Bernstein *et al.* (1995 ▶).
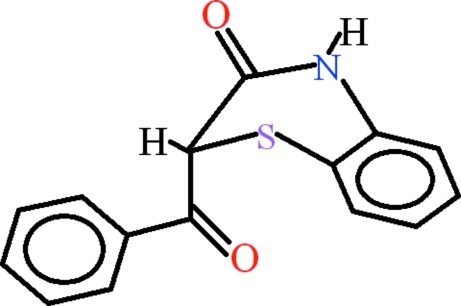


## Experimental

### 

#### Crystal data

C_15_H_11_NO_2_S
*M* *_r_* = 269.31
Monoclinic, 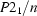

*a* = 9.1323 (3) Å
*b* = 15.2893 (4) Å
*c* = 10.5214 (4) Å
β = 114.669 (1)°
*V* = 1334.99 (8) Å^3^
*Z* = 4
Mo *K*α radiation
μ = 0.24 mm^−1^
*T* = 296 K
0.25 × 0.20 × 0.10 mm

#### Data collection

Bruker Kappa APEXII CCD diffractometer
Absorption correction: multi-scan (*SADABS*; Bruker, 2005 ▶) *T*_min_ = 0.939, *T*_max_ = 0.950
10387 measured reflections
2399 independent reflections
2064 reflections with *I* > 2σ(*I*)
*R*_int_ = 0.023

#### Refinement

*R*[*F* ^2^ > 2σ(*F* ^2^)] = 0.038
*wR*(*F* ^2^) = 0.103
*S* = 1.04
2399 reflections
172 parameters
H-atom parameters constrained
Δρ_max_ = 0.29 e Å^−3^
Δρ_min_ = −0.41 e Å^−3^

### 

Data collection: *APEX2* (Bruker, 2009 ▶); cell refinement: *SAINT* (Bruker, 2009 ▶); data reduction: *SAINT*; program(s) used to solve structure: *SHELXS97* (Sheldrick, 2008 ▶); program(s) used to refine structure: *SHELXL97* (Sheldrick, 2008 ▶); molecular graphics: *ORTEP-3 for Windows* (Farrugia, 1997 ▶) and *PLATON* (Spek, 2009 ▶); software used to prepare material for publication: *WinGX* (Farrugia, 1999 ▶) and *PLATON*.

## Supplementary Material

Crystal structure: contains datablocks global, I. DOI: 10.1107/S1600536810029582/si2280sup1.cif

Structure factors: contains datablocks I. DOI: 10.1107/S1600536810029582/si2280Isup2.hkl

Additional supplementary materials:  crystallographic information; 3D view; checkCIF report

## Figures and Tables

**Table 1:** Hydrogen-bond geometry (Å, °)

*D*—H⋯*A*	*D*—H	H⋯*A*	*D*⋯*A*	*D*—H⋯*A*
N1—H1⋯O2^i^	0.86	1.98	2.8383 (19)	179
C2—H2⋯O1^ii^	0.93	2.55	3.453 (2)	165
C8—H8⋯O1^ii^	0.98	2.36	3.170 (2)	140

Symmetry codes: (i) 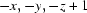; (ii) 
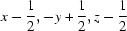
.

**Fig. 1.**
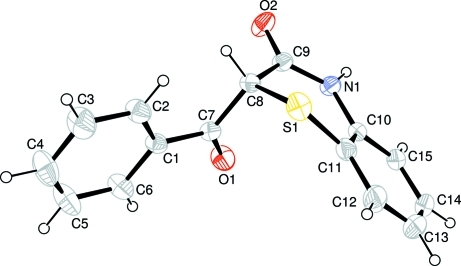
View of the title compound with the atom numbering scheme. The thermal ellipsoids are drawn at the 30% probability level. H-atoms are shown by small circles of arbitrary radii.

**Fig. 2.**
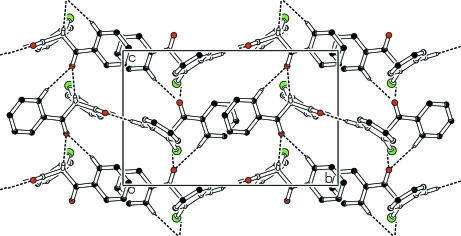
The partial packing (PLATON; Spek, 2009) which shows that molecules form dimers which are interlinked in the form of polymeric sheets. The H-atoms not involved in H-bodings are omitted for clarity.

## supplementary crystallographic information

### Comment

Benzothiazin molecules exhibit a broad spectrum of biological activity such as antifungal (Güven *et al.*, 2007) and antibacterial (Nofal *et al.*, 2002) properties. Our group is engaged in preparation and evaluation of biological activities of such type of compounds.

The crystal structures of (II) *i.e.*, 2-(2-(4-bromophenyl)-2-oxoethyl)-4*H*-benzo-1,4-thiazin-3-one (Beryozkina *et al.*, 2004), (III) 2-(4-chlorobenzoylmethyl)-2*H*-1,4-benzothiazin-3(4*H*)-one (Zhang *et al.*, 2008) and (IV) (±)-2-(hydroxy(4-methoxyphenyl)methyl)-2*H*-1,4-benzothiazin- 3(4*H*)-one monohydrate (Kumaradhas & Nirmala *et al.*, 1997) have been published which are related to title compound (I), (Fig. 1).

In (I), the benzene rings A (C1—C6) and B (C10—C15) are planar with r. m. s. deviations of 0.0045 and 0.0084 Å, respectively. The dihedral angle between A/B is 80.35 (7)°. The heterocyclic six membered ring C (C8/C9/N1/C10/C11/S1) fused with phenyl ring group B is not planar. The confirmation of this ring may be described by the puckering parameters (Cremer & Pople, 1975): Q = 0.5308 (15) Å, θ = 63.11 (18)°, φ = 23.5 (2)°. The molecules are stabilized in the form of dimers (Fig. 2) due to N—H···O type of H-bondings (Table 1) with *R*_2_^2^(8) ring motifs (Bernstein *et al.*, 1995). The dimers are interlinked in the form of polymeric sheets extending parallel to *bc*-plane due to C—H···O type of H-bondings (Table 1, Fig. 2) and complete *R*_2_^1^(7) ring motifs. There exist π–π interaction between the centroids of carbonyl containing phenyl rings at a separation of 3.9187 (15) Å [symmetry code: -*x*, 1 - *y*, 1 - *z*].

### Experimental

2-Aminothiophenol (0.01 *M*, 1.08 ml) and ethyl benzoyl acetate (0.01 *M*, 1.73) were added to 5 ml dimethylsulfoxide. Resulting mixture was refluxed for 1 h and left overnight at room temperature. The separated solid was filtered, washed with petroleum ether and recrystallized with methanol to affoard light yellow plates.

### Refinement

All H-atoms were positioned geometrically (N—H = 0.86, C–H = 0.93–0.98 Å) and refined as riding with *U*_iso_(H) = *xU*_eq_(C, N), where *x* = 1.2 for all H-atoms.

### Crystal data

**Table:**

C_15_H_11_NO_2_S	*F*(000) = 560
*M**_r_* = 269.31	*D*_x_ = 1.340 Mg m^−^^3^
Monoclinic, *P*2_1_/*n*	Mo *K*α radiation, λ = 0.71073 Å
Hall symbol: -P 2yn	Cell parameters from 2064 reflections
*a* = 9.1323 (3) Å	θ = 3.4–25.3°
*b* = 15.2893 (4) Å	µ = 0.24 mm^−^^1^
*c* = 10.5214 (4) Å	*T* = 296 K
β = 114.669 (1)°	Plate, light yellow
*V* = 1334.99 (8) Å^3^	0.25 × 0.20 × 0.10 mm
*Z* = 4	

### Data collection

**Table:**

Bruker Kappa APEXII CCD diffractometer	2399 independent reflections
Radiation source: fine-focus sealed tube	2064 reflections with *I* > 2σ(*I*)
graphite	*R*_int_ = 0.023
Detector resolution: 8.10 pixels mm^-1^	θ_max_ = 25.3°, θ_min_ = 3.4°
ω scans	*h* = −10→10
Absorption correction: multi-scan (*SADABS*; Bruker, 2005)	*k* = −18→18
*T*_min_ = 0.939, *T*_max_ = 0.950	*l* = −12→12
10387 measured reflections	

### Refinement

**Table:**

Refinement on *F*^2^	Primary atom site location: structure-invariant direct methods
Least-squares matrix: full	Secondary atom site location: difference Fourier map
*R*[*F*^2^ > 2σ(*F*^2^)] = 0.038	Hydrogen site location: inferred from neighbouring sites
*wR*(*F*^2^) = 0.103	H-atom parameters constrained
*S* = 1.04	*w* = 1/[σ^2^(*F*_o_^2^) + (0.046*P*)^2^ + 0.5415*P*] where *P* = (*F*_o_^2^ + 2*F*_c_^2^)/3
2399 reflections	(Δ/σ)_max_ < 0.001
172 parameters	Δρ_max_ = 0.29 e Å^−^^3^
0 restraints	Δρ_min_ = −0.41 e Å^−^^3^

### Special details

**Table:**

Geometry. Bond distances, angles *etc*. have been calculated using the rounded fractional coordinates. All su's are estimated from the variances of the (full) variance-covariance matrix. The cell e.s.d.'s are taken into account in the estimation of distances, angles and torsion angles
Refinement. Refinement of *F*^2^ against ALL reflections. The weighted *R*-factor *wR* and goodness of fit *S* are based on *F*^2^, conventional *R*-factors *R* are based on *F*, with *F* set to zero for negative *F*^2^. The threshold expression of *F*^2^ > σ(*F*^2^) is used only for calculating *R*-factors(gt) *etc*. and is not relevant to the choice of reflections for refinement. *R*-factors based on *F*^2^ are statistically about twice as large as those based on *F*, and *R*- factors based on ALL data will be even larger.

### Fractional atomic coordinates and isotropic or equivalent isotropic displacement parameters (Å^2^)

**Table:**

	*x*	*y*	*z*	*U*_iso_*/*U*_eq_	
S1	−0.02233 (6)	0.25059 (3)	0.25961 (5)	0.0528 (2)	
O1	0.07429 (16)	0.26350 (9)	0.61431 (13)	0.0569 (4)	
O2	−0.15131 (15)	0.08259 (8)	0.46234 (17)	0.0603 (5)	
N1	0.08715 (16)	0.09439 (9)	0.45062 (15)	0.0419 (4)	
C1	−0.1244 (2)	0.36835 (10)	0.49255 (17)	0.0399 (5)	
C2	−0.2555 (2)	0.39269 (12)	0.3713 (2)	0.0508 (6)	
C3	−0.3331 (3)	0.47100 (14)	0.3657 (3)	0.0720 (8)	
C4	−0.2798 (3)	0.52592 (15)	0.4793 (3)	0.0827 (10)	
C5	−0.1488 (3)	0.50307 (15)	0.5989 (3)	0.0795 (9)	
C6	−0.0712 (3)	0.42488 (13)	0.6065 (2)	0.0584 (7)	
C7	−0.04028 (19)	0.28361 (10)	0.50775 (16)	0.0372 (5)	
C8	−0.09851 (19)	0.21947 (10)	0.38628 (16)	0.0365 (5)	
C9	−0.05515 (19)	0.12647 (10)	0.43619 (18)	0.0401 (5)	
C10	0.2099 (2)	0.13817 (11)	0.42926 (18)	0.0402 (5)	
C11	0.1776 (2)	0.21359 (12)	0.34898 (19)	0.0470 (6)	
C12	0.3019 (3)	0.25556 (14)	0.3316 (3)	0.0678 (9)	
C13	0.4547 (3)	0.22130 (18)	0.3895 (3)	0.0782 (10)	
C14	0.4862 (3)	0.14499 (16)	0.4662 (3)	0.0672 (8)	
C15	0.3646 (2)	0.10353 (13)	0.4873 (2)	0.0505 (6)	
H1	0.10555	0.04056	0.47600	0.0503*	
H2	−0.29116	0.35614	0.29359	0.0609*	
H3	−0.42186	0.48663	0.28466	0.0864*	
H4	−0.33252	0.57860	0.47507	0.0992*	
H5	−0.11211	0.54072	0.67529	0.0952*	
H6	0.01709	0.40971	0.68826	0.0701*	
H8	−0.21632	0.22331	0.34039	0.0439*	
H12	0.28195	0.30724	0.28049	0.0813*	
H13	0.53768	0.24977	0.37689	0.0939*	
H14	0.58965	0.12159	0.50366	0.0807*	
H15	0.38578	0.05252	0.54010	0.0606*	

### Atomic displacement parameters (Å^2^)

**Table:**

	*U*^11^	*U*^22^	*U*^33^	*U*^12^	*U*^13^	*U*^23^
S1	0.0689 (4)	0.0489 (3)	0.0425 (3)	0.0067 (2)	0.0251 (2)	0.0100 (2)
O1	0.0513 (8)	0.0588 (8)	0.0406 (7)	0.0149 (6)	−0.0005 (6)	−0.0002 (6)
O2	0.0459 (7)	0.0373 (7)	0.1062 (12)	0.0049 (6)	0.0402 (8)	0.0184 (7)
N1	0.0378 (7)	0.0277 (7)	0.0595 (9)	0.0014 (6)	0.0196 (7)	0.0057 (6)
C1	0.0436 (9)	0.0314 (8)	0.0425 (9)	−0.0020 (7)	0.0157 (7)	0.0031 (7)
C2	0.0540 (11)	0.0365 (9)	0.0502 (10)	0.0051 (8)	0.0103 (9)	0.0046 (8)
C3	0.0762 (15)	0.0488 (12)	0.0743 (14)	0.0220 (11)	0.0149 (12)	0.0147 (11)
C4	0.112 (2)	0.0438 (12)	0.0907 (18)	0.0277 (13)	0.0407 (16)	0.0041 (12)
C5	0.114 (2)	0.0478 (12)	0.0713 (15)	0.0101 (13)	0.0334 (14)	−0.0125 (11)
C6	0.0721 (13)	0.0466 (11)	0.0477 (10)	0.0033 (10)	0.0163 (10)	−0.0022 (8)
C7	0.0359 (9)	0.0358 (9)	0.0365 (8)	−0.0010 (7)	0.0119 (7)	0.0046 (7)
C8	0.0334 (8)	0.0314 (8)	0.0388 (8)	0.0014 (6)	0.0092 (7)	0.0035 (6)
C9	0.0354 (9)	0.0310 (8)	0.0513 (10)	−0.0004 (7)	0.0154 (7)	0.0032 (7)
C10	0.0412 (9)	0.0362 (9)	0.0468 (9)	−0.0054 (7)	0.0220 (7)	−0.0101 (7)
C11	0.0581 (11)	0.0420 (10)	0.0508 (10)	−0.0057 (8)	0.0326 (9)	−0.0062 (8)
C12	0.0860 (17)	0.0564 (13)	0.0891 (16)	−0.0114 (11)	0.0645 (14)	−0.0042 (11)
C13	0.0770 (16)	0.0757 (16)	0.113 (2)	−0.0253 (13)	0.0704 (16)	−0.0254 (15)
C14	0.0474 (11)	0.0784 (16)	0.0876 (16)	−0.0081 (10)	0.0398 (11)	−0.0316 (13)
C15	0.0437 (10)	0.0492 (10)	0.0607 (11)	0.0002 (8)	0.0239 (9)	−0.0144 (9)

### Geometric parameters (Å, °)

**Table:**

S1—C8	1.8057 (18)	C10—C15	1.389 (3)
S1—C11	1.762 (2)	C10—C11	1.386 (2)
O1—C7	1.211 (2)	C11—C12	1.381 (3)
O2—C9	1.224 (2)	C12—C13	1.372 (4)
N1—C9	1.337 (2)	C13—C14	1.379 (4)
N1—C10	1.402 (2)	C14—C15	1.375 (4)
N1—H1	0.8600	C2—H2	0.9300
C1—C6	1.391 (3)	C3—H3	0.9300
C1—C7	1.481 (2)	C4—H4	0.9300
C1—C2	1.387 (3)	C5—H5	0.9300
C2—C3	1.380 (3)	C6—H6	0.9300
C3—C4	1.373 (4)	C8—H8	0.9800
C4—C5	1.371 (4)	C12—H12	0.9300
C5—C6	1.375 (3)	C13—H13	0.9300
C7—C8	1.520 (2)	C14—H14	0.9300
C8—C9	1.510 (2)	C15—H15	0.9300
			
S1···O1	3.4635 (14)	C11···O1	3.386 (2)
S1···N1	3.0108 (15)	C14···S1^ii^	3.505 (3)
S1···C2	3.567 (2)	C15···S1^ii^	3.432 (2)
S1···C15^i^	3.432 (2)	C2···H8	2.6500
S1···O1^i^	3.3545 (16)	C2···H13^iv^	2.9000
S1···C14^i^	3.505 (3)	C8···H2	2.6400
S1···H2	3.0800	C9···H1^iii^	2.8200
O1···S1	3.4635 (14)	C9···H3^v^	3.1000
O1···N1	3.136 (2)	H1···H15	2.3700
O1···C10	3.318 (2)	H1···O2^iii^	1.9800
O1···C11	3.386 (2)	H1···C9^iii^	2.8200
O1···S1^ii^	3.3545 (16)	H1···H1^iii^	2.5100
O1···C8^ii^	3.170 (2)	H2···S1	3.0800
O2···N1^iii^	2.8383 (19)	H2···C8	2.6400
O1···H6	2.4900	H2···H8	2.1300
O1···H8^ii^	2.3600	H2···O1^i^	2.5500
O1···H2^ii^	2.5500	H3···N1^vi^	2.8300
O2···H14^iv^	2.6500	H3···C9^vi^	3.1000
O2···H1^iii^	1.9800	H6···O1	2.4900
N1···S1	3.0108 (15)	H8···C2	2.6500
N1···O1	3.136 (2)	H8···H2	2.1300
N1···O2^iii^	2.8383 (19)	H8···H13^iv^	2.4700
N1···H3^v^	2.8300	H8···O1^i^	2.3600
C2···S1	3.567 (2)	H13···C2^vii^	2.9000
C7···C10	3.524 (3)	H13···H8^vii^	2.4700
C8···O1^i^	3.170 (2)	H14···O2^vii^	2.6500
C10···O1	3.318 (2)	H15···H1	2.3700
C10···C7	3.524 (3)		
			
C8—S1—C11	98.87 (9)	S1—C11—C12	120.40 (16)
C9—N1—C10	127.66 (14)	C11—C12—C13	120.2 (2)
C10—N1—H1	116.00	C12—C13—C14	120.5 (3)
C9—N1—H1	116.00	C13—C14—C15	120.0 (3)
C2—C1—C7	122.95 (15)	C10—C15—C14	119.7 (2)
C2—C1—C6	118.88 (17)	C1—C2—H2	120.00
C6—C1—C7	118.16 (16)	C3—C2—H2	120.00
C1—C2—C3	120.18 (19)	C2—C3—H3	120.00
C2—C3—C4	120.3 (2)	C4—C3—H3	120.00
C3—C4—C5	120.0 (2)	C3—C4—H4	120.00
C4—C5—C6	120.4 (2)	C5—C4—H4	120.00
C1—C6—C5	120.3 (2)	C4—C5—H5	120.00
O1—C7—C1	122.07 (15)	C6—C5—H5	120.00
O1—C7—C8	118.58 (15)	C1—C6—H6	120.00
C1—C7—C8	119.35 (14)	C5—C6—H6	120.00
S1—C8—C7	110.07 (12)	S1—C8—H8	108.00
C7—C8—C9	111.48 (13)	C7—C8—H8	108.00
S1—C8—C9	112.26 (12)	C9—C8—H8	108.00
O2—C9—C8	119.05 (16)	C11—C12—H12	120.00
N1—C9—C8	119.09 (15)	C13—C12—H12	120.00
O2—C9—N1	121.86 (15)	C12—C13—H13	120.00
C11—C10—C15	120.25 (18)	C14—C13—H13	120.00
N1—C10—C11	120.91 (17)	C13—C14—H14	120.00
N1—C10—C15	118.82 (16)	C15—C14—H14	120.00
C10—C11—C12	119.31 (19)	C10—C15—H15	120.00
S1—C11—C10	120.12 (15)	C14—C15—H15	120.00
			
C11—S1—C8—C7	76.58 (13)	O1—C7—C8—S1	−99.48 (17)
C11—S1—C8—C9	−48.23 (14)	O1—C7—C8—C9	25.8 (2)
C8—S1—C11—C10	35.41 (16)	C1—C7—C8—S1	80.43 (18)
C8—S1—C11—C12	−149.38 (18)	C1—C7—C8—C9	−154.32 (16)
C10—N1—C9—O2	−177.54 (17)	S1—C8—C9—O2	−144.64 (15)
C10—N1—C9—C8	2.4 (3)	S1—C8—C9—N1	35.38 (19)
C9—N1—C10—C11	−20.1 (3)	C7—C8—C9—O2	91.3 (2)
C9—N1—C10—C15	161.39 (17)	C7—C8—C9—N1	−88.65 (19)
C6—C1—C2—C3	−1.3 (3)	N1—C10—C11—S1	−5.5 (2)
C7—C1—C2—C3	177.2 (2)	N1—C10—C11—C12	179.22 (19)
C2—C1—C6—C5	0.7 (3)	C15—C10—C11—S1	172.94 (14)
C7—C1—C6—C5	−177.9 (2)	C15—C10—C11—C12	−2.3 (3)
C2—C1—C7—O1	179.82 (18)	N1—C10—C15—C14	179.28 (19)
C2—C1—C7—C8	−0.1 (3)	C11—C10—C15—C14	0.8 (3)
C6—C1—C7—O1	−1.7 (3)	S1—C11—C12—C13	−173.2 (2)
C6—C1—C7—C8	178.41 (19)	C10—C11—C12—C13	2.1 (3)
C1—C2—C3—C4	0.9 (4)	C11—C12—C13—C14	−0.4 (4)
C2—C3—C4—C5	0.1 (4)	C12—C13—C14—C15	−1.2 (4)
C3—C4—C5—C6	−0.7 (4)	C13—C14—C15—C10	1.0 (4)
C4—C5—C6—C1	0.3 (4)		

Symmetry codes: (i) *x*−1/2, −*y*+1/2, *z*−1/2; (ii) *x*+1/2, −*y*+1/2, *z*+1/2; (iii) −*x*, −*y*, −*z*+1; (iv) *x*−1, *y*, *z*; (v) −*x*−1/2, *y*−1/2, −*z*+1/2; (vi) −*x*−1/2, *y*+1/2, −*z*+1/2; (vii) *x*+1, *y*, *z*.

### Hydrogen-bond geometry (Å, °)

**Table:**

*D*—H···*A*	*D*—H	H···*A*	*D*···*A*	*D*—H···*A*
N1—H1···O2^iii^	0.86	1.98	2.8383 (19)	179
C2—H2···O1^i^	0.93	2.55	3.453 (2)	165
C8—H8···O1^i^	0.98	2.36	3.170 (2)	140

Symmetry codes: (iii) −*x*, −*y*, −*z*+1; (i) *x*−1/2, −*y*+1/2, *z*−1/2.
